# T87. TOWARD DEVELOPING CLINICAL CUTOFF VALUES FOR THE BECK COGNITIVE INSIGHT SCALE

**DOI:** 10.1093/schbul/sby016.363

**Published:** 2018-04-01

**Authors:** Danielle Penney, Genevieve Sauve, Ashok Malla, Ridha Joober, Martin Lepage

**Affiliations:** 1 Douglas Mental Health University Institute; 2 McGill University

## Abstract

**Background:**

Cognitive insight represents the ability to question and criticize the validity of one’s beliefs, to recognize when beliefs may be faulty, and to then rely on external feedback to make correct assessments of a situation. Cognitive insight is characteristically impaired in persons with schizophrenia and related psychoses. The Beck Cognitive Insight Scale (BCIS) is the most widely used tool to assess cognitive insight, yet there is no consensus regarding clinical cutoff values. Cognitive insight is predictive of better response to psychosocial treatment and the ability to accept critical feedback from treatment teams, thus cutoffs are an important next step needed to facilitate the clinical interpretation of the BCIS. Some studies have attempted to develop diagnostic cutoffs, yet no study has proposed clinical cutoffs to differentiate levels of cognitive insight between patients with schizophrenia.

**Methods:**

Three hundred and eighty-five English or French-speaking patients with a schizophrenia spectrum disorder (203 first-episode and 182 multiple-episode psychosis patients) and 185 healthy controls completed a battery of clinical and neuropsychological tests, including the BCIS. Patients and controls were matched on age, sex, level of education, and socio-economic-status. Correlations were calculated between the composite index and previously identified correlates of cognitive insight. Variables significantly correlating with the BCIS composite index were then included in a clustering analysis to classify patients according to their clinical profile. Two clinical profiles representing low and high cognitive insight were identified, and were based on global functioning and IQ. Composite index scores at the 33rd percentile in the low cognitive insight cluster and the 66th percentile in the high cognitive insight cluster were calculated.

**Results:**

Functioning and IQ significantly correlated with the BCIS composite index and were included in a clustering analysis, using a pre-determined number of two clusters. Independent samples t-tests revealed that the 2 clusters differed significantly on the BCIS self-reflectiveness score (t(372) = -3.93, p < .001) and on the composite index (t(372) = -3.17, p = .002). There was no difference between clusters on self-certainty (t(372) = .31, p = .76). Patients in cluster A had a mean SR, SC, and composite index of 12.65 (SD = 4.3, Range = 2 to 26), 7.78 (SD = 3.3, Range = 0 to 18) and 4.87 (SD = 5.8, Range = -11 to 20), respectively, while mean scores for patients in cluster B were 15.11 (SD = 4.1, Range = 3 to 25), 7.64 (SD = 2.9, Range = 1 to 15) and 7.47 (SD = 4.8, Range = -3 to 22). In cluster A, the values of the 33rd and 66th percentiles were 2.6 and 7 respectfully. In cluster B, these values were 5 and 9. We are proposing that 33% of patients with the lowest composite index scores in cluster A represent those with low cognitive insight. Accordingly, 33% of patients with the highest composite index scores classified in cluster B represent those with high cognitive insight. Low cognitive insight is thus represented by a score of 3 or below, borderline scores range from 4 to 9, and high cognitive insight is represented by a score of 10 or above.

**Discussion:**

We proposed clinical cutoffs for the BCIS with a theoretical basis anchored in patient clinical profiles (functioning and IQ). Clinical cutoffs will facilitate and better orient treatment teams in the clinical interpretation of the BCIS and ergo to patients’ level of cognitive insight. The development of such cutoffs will help to reduce heterogeneity in psychosocial group intervention, will facilitate interventions aimed at increasing cognitive insight, and improve communication between patients and their treatment teams.

